# Brain state dynamics differ between eyes open and eyes closed rest

**DOI:** 10.1002/hbm.26746

**Published:** 2024-07-11

**Authors:** Brandon T. Ingram, Stephen D. Mayhew, Andrew P. Bagshaw

**Affiliations:** ^1^ Centre for Human Brain Health, School of Psychology University of Birmingham Birmingham UK; ^2^ Institute of Health and Neurodevelopment School of Psychology, Aston University Birmingham UK

**Keywords:** EEG‐fMRI, eyes closed, eyes open, hidden Markov model, resting‐state

## Abstract

The human brain exhibits spatio‐temporally complex activity even in the absence of external stimuli, cycling through recurring patterns of activity known as brain states. Thus far, brain state analysis has primarily been restricted to unimodal neuroimaging data sets, resulting in a limited definition of state and a poor understanding of the spatial and temporal relationships between states identified from different modalities. Here, we applied hidden Markov model (HMM) to concurrent electroencephalography‐functional magnetic resonance imaging (EEG‐fMRI) eyes open (EO) and eyes closed (EC) resting‐state data, training models on the EEG and fMRI data separately, and evaluated the models' ability to distinguish dynamics between the two rest conditions. Additionally, we employed a general linear model approach to identify the BOLD correlates of the EEG‐defined states to investigate whether the fMRI data could be used to improve the spatial definition of the EEG states. Finally, we performed a sliding window‐based analysis on the state time courses to identify slower changes in the temporal dynamics, and then correlated these time courses across modalities. We found that both models could identify expected changes during EC rest compared to EO rest, with the fMRI model identifying changes in the activity and functional connectivity of visual and attention resting‐state networks, while the EEG model correctly identified the canonical increase in alpha upon eye closure. In addition, by using the fMRI data, it was possible to infer the spatial properties of the EEG states, resulting in BOLD correlation maps resembling canonical alpha‐BOLD correlations. Finally, the sliding window analysis revealed unique fractional occupancy dynamics for states from both models, with a selection of states showing strong temporal correlations across modalities. Overall, this study highlights the efficacy of using HMMs for brain state analysis, confirms that multimodal data can be used to provide more in‐depth definitions of state and demonstrates that states defined across different modalities show similar temporal dynamics.

## Graphical abstract

We apply hidden Markov model to concurrently collected electroencephalography‐functional magnetic resonance imaging (EEG‐fMRI) resting‐state data, to investigate differences in brain states between eyes open (EO) and eyes closed (EC) resting states. The results showed significant differences in the brain state dynamics of both EEG and fMRI states between EO and EC rest.

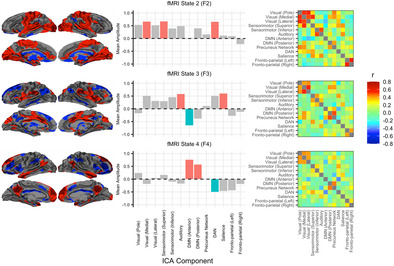

**Practitioner Points**

- Hidden Markov model (HMM) reveals brain states in electroencephalography (EEG) and functional magnetic resonance imaging (fMRI) data that differ between eyes open and eyes closed (EC) resting states.
- EEG HMM identified a high alpha power state with significant BOLD correlates resembling previously identified alpha‐BOLD correlations, despite only being active for ~15% of the EC runs.
- State temporal dynamics exhibited strong temporal correlations across neuroimaging modalities.

## 1 INTRODUCTION

During periods of waking rest, the human brain continues to exhibit spatiotemporally complex activity despite the reduction in external stimuli. Commonly referred to as resting‐state data, subjects are instructed to relax throughout the duration of a recording (e.g., functional magnetic resonance imaging [fMRI] or E/MEG), without any task being present. The analysis of such data has led to the discovery of various resting‐state networks, which have since been shown to persist throughout both rest and task conditions (Biswal et al., 1995; Smith et al., 2009). These have been associated with numerous cognitive processes (Buckner et al., 2008; Corbetta & Shulman, 2002; Vossel et al., 2014), as well as exhibiting differences between clinical and control populations, and could potentially serve as biomarkers for neurological and mental health conditions (Buckner et al., 2008; Calhoun et al., 2009; Greicius et al., 2004; Rosazza & Minati, 2011). Conventionally, these networks have been identified by functional connectivity (FC) based approaches, in which the statistical temporal correlation between the neural activity of multiple brain regions is calculated. Resting‐state networks are then defined as a group of regions showing strong FC with each other. These approaches have generally been applied across the entire duration of a recording lasting several minutes, providing a summary measure of the FC between regions over this timescale. Although this method has proved useful, it assumes that the FC between regions is static/stationary, or in other words, that the relationship between brain regions does not change over the duration of the recording. Many studies have demonstrated that this is clearly an oversimplification of the complexity of the brain's activity.

Dynamic approaches, such as sliding window analyses (Allen et al., 2014), leading eigenvector dynamic analysis (Cabral et al., 2017), and temporal independent component analysis (ICA) (Smith et al., 2012), have all demonstrated that FC relationships are not static/stationary; and that the temporal correlations, both between network nodes and between different networks, vary over time. Furthermore, these changes in network FC are non‐random, with specific patterns of activity reoccurring throughout the duration of recordings (Allen et al., 2014; Sakoğlu et al., 2010). This has most commonly been demonstrated using sliding window‐based analyses, followed by k‐means clustering, resulting in a set of reoccurring FC patterns, commonly referred to as connectivity, or brain, ‘states’. Once identified, it is then possible to calculate various temporal metrics for each state, such as the average time a subject spends within each state (state lifetimes), or the frequency at which subjects switch between states (switching rates). Collectively, this has led to the proposal of the chronnectome (Calhoun et al., 2014) or the dynome (Kopell et al., 2014), which aims to provide a comprehensive characterisation of brain FC and dynamics (Calhoun et al., 2014). Such methods have also shown potential clinical application, with people with schizophrenia exhibiting altered connectivity states (Sakoğlu et al., 2010) and state temporal metrics (Damaraju et al., 2014) relative to control participants.

However, sliding window‐based analyses have recently been criticised due to the requirement to arbitrarily select parameters, such as the window length and offset (Hindriks et al., 2016; Laumann et al., 2017; Leonardi & Van De Ville, 2015; Zalesky & Breakspear, 2015). Additionally, simulations have shown that the method's ability to detect state transitions and state durations is significantly impacted by the choice of window length (Shakil et al., 2016). An alternative approach is to identify and model brain state dynamics with a HMM (Baker et al., 2014; Vidaurre et al., 2016). Unlike sliding windows, HMM aims to decompose the data into a dynamic sequence of distinct states in a data‐driven manner, therefore avoiding the need to specify a window length and offset.

However, current research has exclusively focused on quantifying brain states in one neuroimaging modality, and very little is known about how brain states compare across modalities, or the relationships between neural and haemodynamic brain states. This limits their wider interpretation. Furthermore, the definition of each state is restricted by the advantages and disadvantages of each neuroimaging modality. Ideally, brain states would be defined with both high spatial and temporal resolution. This would best enable monitoring of dynamic activity patterns and regional interactions for applications such as studying recruitment during task performance and its relation to behaviour; or spontaneous alterations during changes in awareness or consciousness. Although fMRI can provide millimetre spatial resolution recording of both cortical and subcortical brain activity, it is limited temporally as a result of the sluggish nature of the BOLD signal (Buxton et al., 1998). Conversely, electrophysiological measures such as E/MEG provide millisecond temporal resolution and a direct measure of neural activity compared to the BOLD signal. They also allow brain activity to be analysed from a spectral perspective, for example, studying specific frequency bands rather than the integration of all underlying local activity represented by the BOLD signal. However, the spatial resolution and sensitivity to subcortical structures of E/MEG are greatly reduced compared to fMRI due to volume conduction.

One potential method to achieve high spatio‐temporal resolution is to employ a multimodal approach, recording electroencephalography (EEG) and fMRI data concurrently. This approach allows for the models to be trained on each modality while using the other to better inform the spatial or temporal properties of the identified states. For example, Hunyadi et al. (2019) applied HMM to concurrent EEG‐fMRI data, defining states by changes in power within the EEG data, and using the state time courses to obtain spatial maps for each state via a general linear model (GLM) analysis of the fMRI data (Hunyadi et al., 2019). To date however, there has been no explicit attempt to define EEG and fMRI brain states from concurrently recorded data and to study the cross‐modal relationships in their properties under a behavioural manipulation.

One of the simplest and most fundamental manipulations of brain activity that can be applied is eye closure. Within EEG, there is a robust and significant increase in alpha power upon eye closure (Barry et al., 2007; Boytsova & Danko, 2010; Li, 2010). Similarly, fMRI studies have found that eye closure induces changes in BOLD activity and FC across visual (Patriat et al., 2013), auditory (Marx et al., 2003; Patriat et al., 2013; Zou et al., 2015), somatosensory (Liang et al., 2014; Yang et al., 2007) and insula cortices (Goldman et al., 2002), as well as the default mode (Jao et al., 2013) and dorsal attention networks (DANs) (Wang et al., 2016; Zhang et al., 2015). Concurrent EEG‐fMRI studies have identified correlations between changes in resting‐state alpha power and the BOLD signal in these brain regions, both using static (de Munck et al., 2007; Goldman et al., 2002; Laufs et al., 2006) and dynamic (Mayhew & Bagshaw, 2017) analysis methods. However, a deeper understanding is required of dynamic and spontaneous alpha‐BOLD relationships, and how they vary with the manipulation of awareness and attention, which can be provided by adopting an analysis of fluctuations in EEG and fMRI brain‐states and an investigation of the relationships between the activity of states defined from the two modalities.

Therefore, this study used HMM analysis of concurrent EEG‐fMRI resting‐state data and compared brain state dynamics between periods of eyes open (EO) and eyes closed (EC) rest. HMMs were trained separately on each modality to allow comparison with previous EEG and fMRI literature. We quantified the changes in brain state dynamics in each of the two modalities, as well as using a GLM to identify the BOLD correlates of EEG states. Furthermore, we employed a sliding window‐based analysis to investigate slower temporal dynamics within each model and then compared them to identify relationships in state dynamics across modalities. The primary goal of this was to further identify differences in the state metrics between the EO and EC conditions, and compare these differences with those found within the literature. Additionally, we aimed to evaluate whether concurrently collected fMRI data could be used to improve the spatial definition of the EEG‐defined states. Finally, we aimed to investigate the temporal relationships of states across modalities.

## 2 METHODS

### 2.1 Participants

A total of 21 healthy participants (11 female, age range = 21–34 years, mean age = 25.8 years) were recruited via opportunity sampling at the University of Birmingham. Ethical approval for the study was given by the Research Ethics Committee of the University of Birmingham, and all participants gave informed consent before participation. No participants were excluded from the fMRI analysis; however, three participants were removed from the EEG analysis due to data corruption.

### 2.2 EEG‐fMRI data acquisition

Concurrent EEG‐fMRI data were acquired on a 3T Philips Achieva scanner using a 32‐channel SENSE receive head coil and 64‐channel EEG system (MR‐plus amplifiers, Brain Products, Germany) at the Birmingham University Imaging Centre (BUIC). Four resting‐state BOLD fMRI scans were acquired using a whole‐brain gradient echo EPI sequence (TR = 2000 ms, TE = 35 ms, flip angle = 80°, voxel size = 3 × 3 × 4 mm^3^, FOV = 240 × 128 × 240 mm^3^ and slices = 32).

Participants were instructed to either keep their EO or EC for the duration of one scan, alternating after each scan (starting with EO). Two scans were acquired for each of the EO and EC conditions. EO and EC were not alternated within a scan. Each resting‐state scan contained 300 whole brain volumes, resulting in 10 min per scan. In total, 1200 volumes were acquired per participant in a task acquisition time of ~40 min. A high resolution (1 mm isotropic MPRAGE with TR = 2000 ms, TE = 2 ms, TI = 880 ms, flip angle = 8° and FOV 256 × 256) T1‐weighted anatomical image was acquired to facilitate alignment to MNI152 space, resulting in a total scan time of ~45 min.

EEG data were acquired via 63 (including reference and ground) Ag/AgCl MR‐compatible scalp electrodes, positioned in accordance with the 10‐20 system, with the remaining electrode recording the subject's electrocardiogram (ECG) from the clavicle (EasyCap, Brain Products, Germany). Electrode impedances were kept below 20 kΩ. EEG was recorded using Brain Vision Recorder (Brain Products, Germany) at a sampling rate of 5 kHz, alongside hardware filters of 0.016–250 Hz. Recordings were synchronised with the MR scanner's internal clock (Syncbox, Brain Products, Munich, Germany) (Mandelkow et al., 2006; Mullinger et al., 2008). The MR scanner's physiological monitoring system was used to record cardiac cycles, via vectorcardiogram (VCG) (Mullinger et al., 2008).

### 2.3 fMRI preprocessing

All fMRI analysis was performed within FSL (6.0.1) (Woolrich et al., 2009). Automated brain extraction (Smith, 2002), motion correction (MCFLIRT) (Jenkinson et al., 2002), slice timing correction, spatial smoothing (Gaussian Kernel, FWHM = 6 mm) and high‐pass temporal filtering (>0.01 Hz) were applied to all fMRI data. Transforms to MNI152 (2 mm) standard space were calculated via non‐linear alignment (FLIRT and FNIRT) (Jenkinson & Smith, 2001). Single‐subject ICA (Beckmann & Smith, 2004) and FIX (Griffanti et al., 2014; Salimi‐Khorshidi et al., 2014) (Training Set = Standard.R, threshold = 20) were applied to the data to identify noise components, which were then removed using fsl_regfilt. Cleaned data were transformed to standard space using the applywarp command with the previously calculated transformations.

### 2.4 fMRI spatial ICA

**FIGURE 1:**
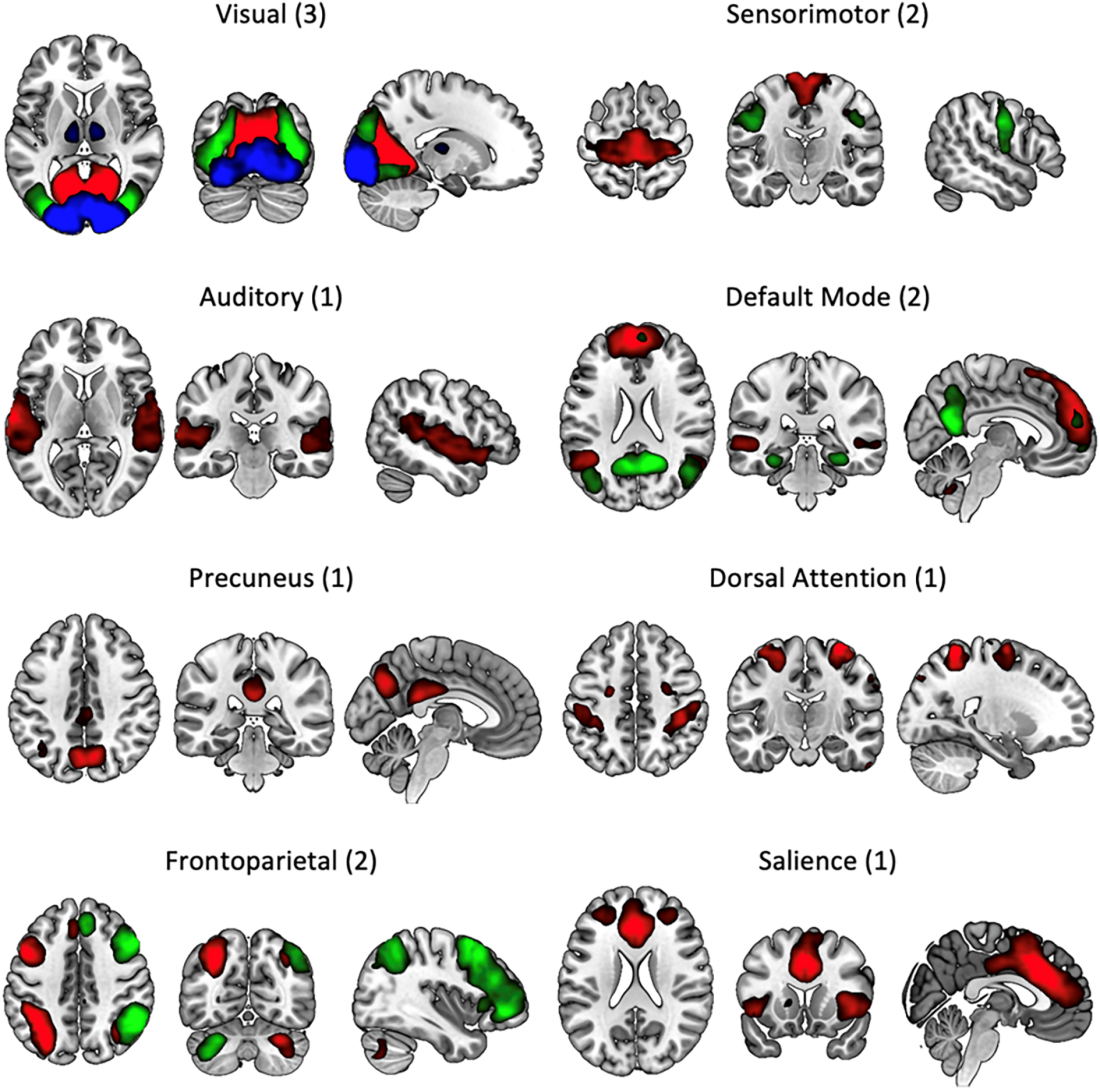
Group spatial ICA results displaying the 13‐network ICs used in subsequent HMM analysis. Numbers in brackets represent the number of ICs that were combined for visualisation in the individual panels. Within a panel, each colour represents a different IC. Images presented using neurological conventions (left = left). HMM, hidden Markov model; ICA, independent component analysis.

As a data reduction step for the fMRI HMM analysis (Ahrends et al., 2022; Vidaurre, Abeysuriya, et al., 2018; Vidaurre et al., 2017), data were temporally concatenated and decomposed into 25 independent components (ICs) via group spatial ICA (MELODIC). A component dimension of 25 was chosen to acquire ICs containing individual, whole resting‐state networks and to avoid overfitting during the HMM training. Only ICs that had time courses in the lower frequencies and did not spatially overlap with known artefacts (motion, vascular, physiological, susceptibility) or non‐grey matter regions (white matter, CSF) were used in the subsequent analysis, resulting in 13 components being retained (see Figure 1). ICs were visually compared with the FIND lab ICA components (Shirer et al., 2012) to assist network labelling. ICs were found within visual (occipital pole, medial and lateral), auditory, dorsal attention, default mode, precuneus, salience, sensorimotor (superior and inferior) and frontoparietal (FPN) (left and right) networks. Dual regression (Beckmann et al., 2009; Nickerson et al., 2017) was applied to estimate BOLD signal time courses for each network IC, in each scan of each subject.

### 2.5 EEG preprocessing

MR‐gradient artefacts were removed from the data using the average template subtraction method with a sliding window (window length = 41 volumes), as implemented within Brain Vision Analyzer 2.0 (Brain Products, Munich, Germany). Heartbeat events were detected on the VCG and added as markers to the EEG via in‐house MATLAB and Perl scripts. Ballistocardiogram (BCG) artefacts were removed via the optimal basis sets method (principal components = 3) using an in‐house Python script ported from the fMRIB EEGLab plugin (Niazy et al., 2005). Unlike the conventional average artefact subtraction approach (i.e., calculate the average artefact and subtract it), a PCA decomposition is conducted on all gradient artefacts, resulting in a set of optimal basis sets. These basis sets are then fitted to each artefact individually and subtracted, allowing the approach to better account for variability in the BCG artefact. For data reduction and based on the eye closure manipulation being expected to have the largest impact on the alpha band, the EEG analysis focused on the alpha band. This restricted spectral band also optimises the data quality, since both lower (Yan et al., 2010) and higher (Allen et al., 2000) frequencies are contaminated by residual BCG and gradient artefacts, respectively. To isolate alpha band activity and exclude residual MR gradient and BCG artefacts, the data were bandpass filtered between 7 and 13 Hz. Data were then averaged referenced, epoched by the TR (2 s) and down sampled to 40 Hz. FastICA (Hyvarinen, 1999) was conducted to further isolate posterior alpha components. Only components with clear alpha‐power peaks (7–13 Hz) and posterior scalp topographies were retained. On average, two to three posterior alpha components were identified per subject, except for one subject who only had one component. Alpha power components were then retro‐projected to channel space.

### 2.6 GLM analysis investigating alpha‐BOLD relationships

In order to identify which regions of the brain showed a correlation with the posterior alpha rhythm, a GLM analysis was conducted. The GLM analysis is a commonly applied method that utilises multiple linear regression to statistically identify which regions of the brain respond to a specific task or stimulus. In this instance, we used the GLM to identify regions of the brain whose BOLD signal was significantly correlated with the power of the posterior alpha rhythm. As this approach has been commonly applied to EEG‐fMRI previously, it is referred to as the conventional alpha‐BOLD GLM. The alpha power of each TR epoch was calculated via Welch's method for electrodes Oz, O1/2, PO3/4 and POz. The average alpha power was calculated across these channels for each TR epoch, resulting in a continuous alpha time course at the same sampling rate as the fMRI data (Mayhew & Bagshaw, 2017; Mayhew, Hylands‐White, et al., 2013; Mayhew, Ostwald, et al., 2013). The continuous alpha power time courses from each scan per participant were demeaned and normalised to control for differences in maximum alpha power between subjects. The alpha power time courses were then convolved with the FSL double gamma function and used as explanatory variables in a first‐level GLM analysis using FSL FEAT (Woolrich et al., 2001), with contrasts for both the mean positive and negative activations. A second‐level FEAT analysis was conducted to obtain the average EO and EC alpha activation maps per subject, followed by a group‐level FEAT analysis (FLAME1, *Z* threshold = 3.1, cluster *p* threshold = 0.05) (Woolrich et al., 2004) to obtain group average maps for EO and EC, as well as EO > EC and EC > EO contrast map.

### 2.7 Alpha power analysis

The average change in alpha power between the EO and EC conditions was quantified and tested statistically using the alpha power time courses created for the GLM. The means of all EO and EC time points were calculated for each subject. Alpha power values were then compared statistically with a paired *s* test.

### 2.8 Hidden Markov model

The HMM is a statistical model that decomposes time series data into a sequence of states. Each state is defined by an observation model, which can be changed depending on the data type, allowing states to be inferred according to different properties of the data. For example, when the model is applied to fMRI data, a multivariate Gaussian model is typically used, as each state can be parametrised according to the mean activity within a region and its covariance with other regions. Conversely, in E/MEG data, the spectral information is generally richer and is often of more interest than the mean activity of an electrode/region. Thus, more complex observation models are often employed, such as the multivariate autoregressive model and the time‐delayed embedded model, both of which allow states to be defined according to the spectral properties of the data (e.g., frequency and phase). Unlike other dynamic analysis approaches (e.g., sliding window approach), the parameters of the HMM (e.g., the initial state, transition matrix and state time courses) and each state (e.g., means and covariance) can be inferred computationally, thus providing a data‐driven approach for the analysis of brain dynamics.

Within this study we used the publicly available HMM‐MAR toolbox (Vidaurre et al., 2016) to separately train two HMMs: one on the fMRI data and one on the EEG data. For the fMRI HMM, the BOLD signal time courses from each network IC, obtained via dual regression, were concatenated across all runs and participants and used as the inputs to the model. For the EEG HMM, the Hilbert transform of the retro‐projected alpha components from electrodes Oz, O1/2, PO3/4 and POz were concatenated and used as inputs for the model, allowing for changes in alpha power to be modelled. Inputs from both models underwent amplitude standardisation (SD = 1, mean = 0). A multivariate Gaussian model was selected as the observation model for both HMMs. For the fMRI HMM, this meant that each state was defined according to the mean amplitude of each resting‐state network IC, as well as the covariance between resting‐state network ICs. For the EEG HMM, each state was defined according to the mean amplitude of each electrode, and the covariance between them.

Model parameters were inferred based on variational Bayes and the minimisation of variational free energy (Vidaurre et al., 2016). The inference process determines the likelihood of any given state being active at each time point (state probability time course), the starting state of the model (initial state), the likelihood of transitioning from one state to another (transition matrix) and the model parameters of each state (mean vector and covariance matrix). Inference was performed at the group level, resulting in common states being defined across all subjects, whereas the state probability time courses were defined at the subject level.

### 2.9 Model selection

Though the inference process provides a data‐driven method for obtaining the model parameters, there are two main issues with this approach. First, the inference process is stochastic, and will not always provide the same results when run multiple times on the same data. Second, the inference process does not provide the optimum number of states. To circumvent these issues, a model selection process is conducted in which HMMs are trained multiple times to assess the reliability of the inference process, and with different state numbers in order to find the optimum state number. For HMMs run on both EEG and fMRI data, the inference process was conducted multiple times, with the number of states ranging from 2 to 15. Additionally, for each state number, the inference process was repeated five times.

To determine the optimum model, several metrics were calculated to assess both the reliability of the inference process and the model's ability to describe the dynamics of the data. These metrics were: the variational free energy, the maximum fractional occupancy values and repeat reliability. The simplest of these measures is the variational free energy, which is the metric minimised during the inference process, with lower free energy values indicating a better model fit. However, this metric typically decreases with the addition of more states, and therefore is not often used to determine the optimum state number alone. The maximum fractional occupancy values represent the largest percentage of the data time series occupied (e.g., the state is active) by a single state of each run. The distribution of these values is indicative of how well the model describes the dynamics of the data, with higher values indicating that the model is being dominated by a single state, and lower values indicating that the dynamics of the data are being explained by a combination of all of the states. Finally, the repeat reliability was assessed by correlating the state time courses across repeats, giving a measure of model similarity (e.g., higher correlation co‐efficient indicates higher reliability and hence a more stable model).

Based on these measures, the model with the highest number of states while still retaining a low max fractional occupancy, variational free energy and high repeat reliability was selected. Initially, the mean repeat reliability values were plotted to identify the state number in which the reliability began to drop (due to the increase in parameters). The maximum fractional occupancies were then calculated for that model to ensure that the model was accurately describing the dynamics, as well as differentiating between the separate runs (e.g. if one of the models repeats has a lower average maximum fractional occupancy). This process was undertaken independently for the EEG and fMRI data.

Once the optimum model was selected, multiple state metrics were calculated to describe the temporal dynamics of the states. These metrics were fractional occupancy (the percentage of the data each state occupies), the state lifetimes (the duration of each state visit), state interval times (the duration of time between two visits of the same state), and the state switching rate. To calculate these state metrics (including the maximum fractional occupancy used for the model selection), we needed to know when each state was ‘active’ (e.g., a state visit). This could be achieved in two ways: by thresholding the state time courses, or by using the Viterbi path. The former approach works by defining a state as active if its likelihood reaches a threshold. For example, if there is an >80% likelihood of a state being active at a time point, it is deemed as ‘active’. The latter approach relies on the Viterbi algorithm, which estimates the most likely sequence of states given the data, also known as the Viterbi path. Within this study, all state metrics were calculated using the Viterbi Path (Forney, 1973).

### 2.10 Estimating spectra and spatial properties of EEG states

As the EEG states were defined on the Hilbert transform of the EEG data, the states were defined based on changes in power as opposed to spectra. When using wideband data, this would mean that it is not immediately possible to determine whether a change in power was associated with a specific frequency band. In this instance, the data were bandpass filtered, and thus it is clear that changes in power reflect changes in alpha power. However, it still did not provide the specific spectra of each state. Thus, the state spectra of each state were estimated using a state‐wise weighted version of the multi‐taper approach in which periods of the data where a particular state was more likely to be active contribute more to the spectral estimation (Vidaurre et al., 2016). This provided a full spectral definition of each state and provides the spectra of each state for visualisation.

To determine the spatial properties of each EEG state, the state probability time courses from the EEG HMM were used within a fMRI GLM analysis (Hunyadi et al., 2019). The time courses of each state were convolved with a double gamma function, and down‐sampled to the same sampling rate as the TR. A separate first‐level GLM was run for each state per scan, with contrasts for both the positive and negative mean activations. Second‐level FEAT analyses generated average state activation maps for both EO and EC conditions at the subject‐level, by grouping across scans, whereas third‐level FEAT (FLAME1, *Z* threshold = 3.1, cluster p threshold = 0.05) analyses created a group average map for each condition, as well as contrast maps between the two conditions (EO > EC & EC < EO).

### 2.11 State metric analysis

State metrics were statistically compared between the EO and EC conditions using paired *t* tests within both the fMRI HMM and EEG HMM analysis. Bonferroni correction was used to control for multiple comparisons. State lifetimes and interval times were not statistically compared due to being dependent on the number of visits/intervals per condition, often resulting in unequal groups.

### 2.12 Sliding window analysis

To observe slower changes in state dynamics across a single scan, the fractional occupancies were also calculated within a 10‐s sliding window, resulting in a fractional occupancy time course (Baker et al., 2014). These time courses were averaged across subjects to compare temporal trends in state dynamics between the EC and EO conditions. Additionally, we computed the Pearson correlation between the time courses from the EEG and fMRI HMM to compare the temporal relationship between states defined by different neuroimaging modalities.

## 3 RESULTS

### 3.1 Alpha power analysis

**FIGURE 2:**
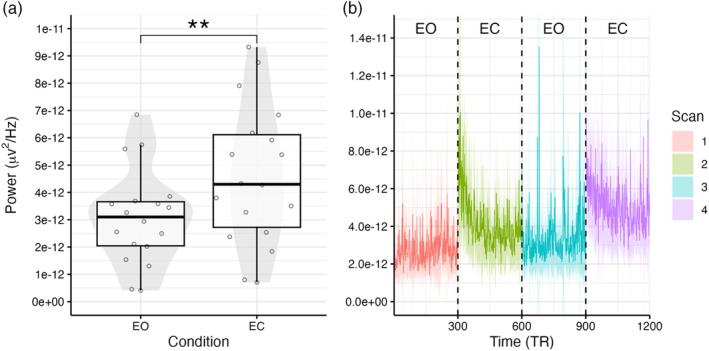
Alpha power compared between the EO and EC conditions. (a) Comparison of the mean alpha power of each condition. ** = *p* < .01. (b) Group‐level alpha power time course at the sampling rate of the fMRI (TR = 2 s). Dashed vertical lines mark the boundaries of each scan. Shaded regions represent 95% confidence intervals between subjects. EC, eyes closed; EEG, electroencephalography; EO, eyes open; fMRI, functional magnetic resonance imaging.

The alpha power analysis found a clear increase in alpha power within the EC condition relative to the EO condition (see Figure 2a). Alpha power showed larger variability between subjects within the EC condition compared to the EO condition. The difference in alpha power was examined statistically using a paired *t* test, which found the difference to be significant with a large effect size (difference = 1.54e−12, 95% confidence interval [CI] [6.52e−13 to 2.43e−12], *t*(17) = 3.66, *p* = .002; Cohen's *d* = 0.86, Cohen's *d* 95% CI: [0.31–1.40]). The mean alpha power time course further revealed that the largest increase in alpha power was observed during the first half of each scan (see Figure 2b). Additionally, the initial increase in alpha power appeared larger within the first EC scan. Conversely, the alpha power in the second EC remained consistently high throughout the duration of the scan, unlike the first EC scan, which eventually returned to a similar amplitude as observed within the EO scans.

### 3.2 Model selection

**FIGURE 3:**
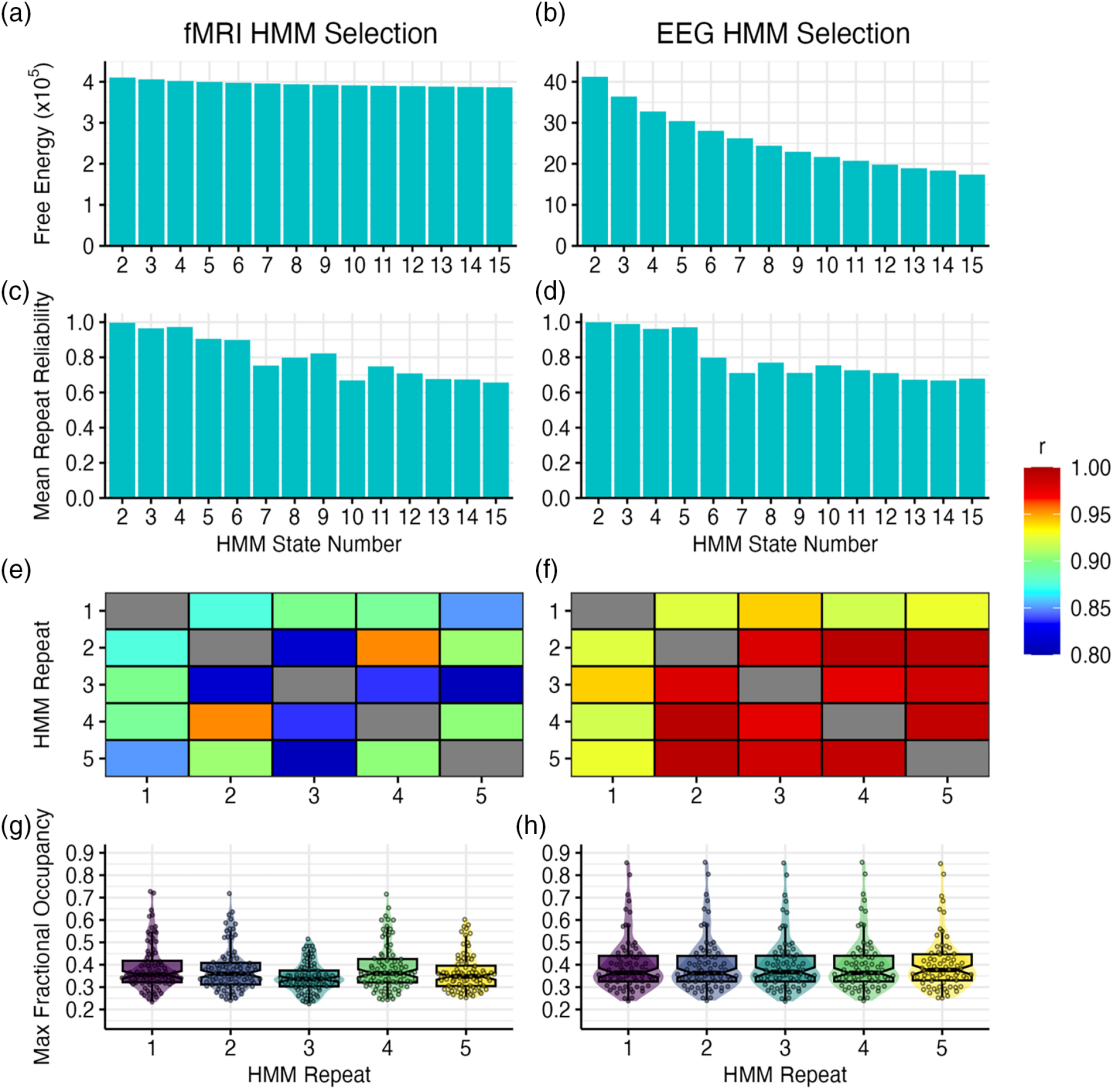
Model selection procedure for fMRI (left column) and EEG (right column) HMMs. (a and b) show the mean free energy for each state number. (c and d) show the mean correlation between repeats for each state number. (e and f) show the correlations between each repeat for state numbers 6 (left) and 5 (right). (g and h) show the max fractional occupancy distributions for each repeat for state numbers 6 (left) and 5 (right). Each point represents the maximum fractional occupancy of each scan. EEG, electroencephalography; fMRI, functional magnetic resonance imaging; HMM, hidden Markov model.

The model selection process aimed to find the highest optimum state number, whereas still possessing a relatively low variational free energy value, high inter‐correlation between repeats and low maximum fractional occupancy values. For both the fMRI and EEG HMM, the variational free energy decreased with the addition of each state (see Figure 3a,b). The mean reliabilities remained consistently high between states two to four within both models, with the reliability dropping at approximately six states in the fMRI model (see Figure 3c), and five states in the EEG model (see Figure 3d). Both state numbers showed high‐inter repeat reliability, with all correlations exceeding 0.80 within the fMRI model (see Figure 3e), and 0.90 within the EEG model (see Figure 3f). Repeat three from the fMRI 6‐state solution possessed the lowest maximum fractional occupancies values and was therefore selected for the analysis (see Figure 3g). Maximum fractional occupancies within the EEG 5‐state solution showed minimal variation, and therefore, the first repeat was selected for analysis (see Figure 3h).

### 3.3 HMM states

**FIGURE 4:**
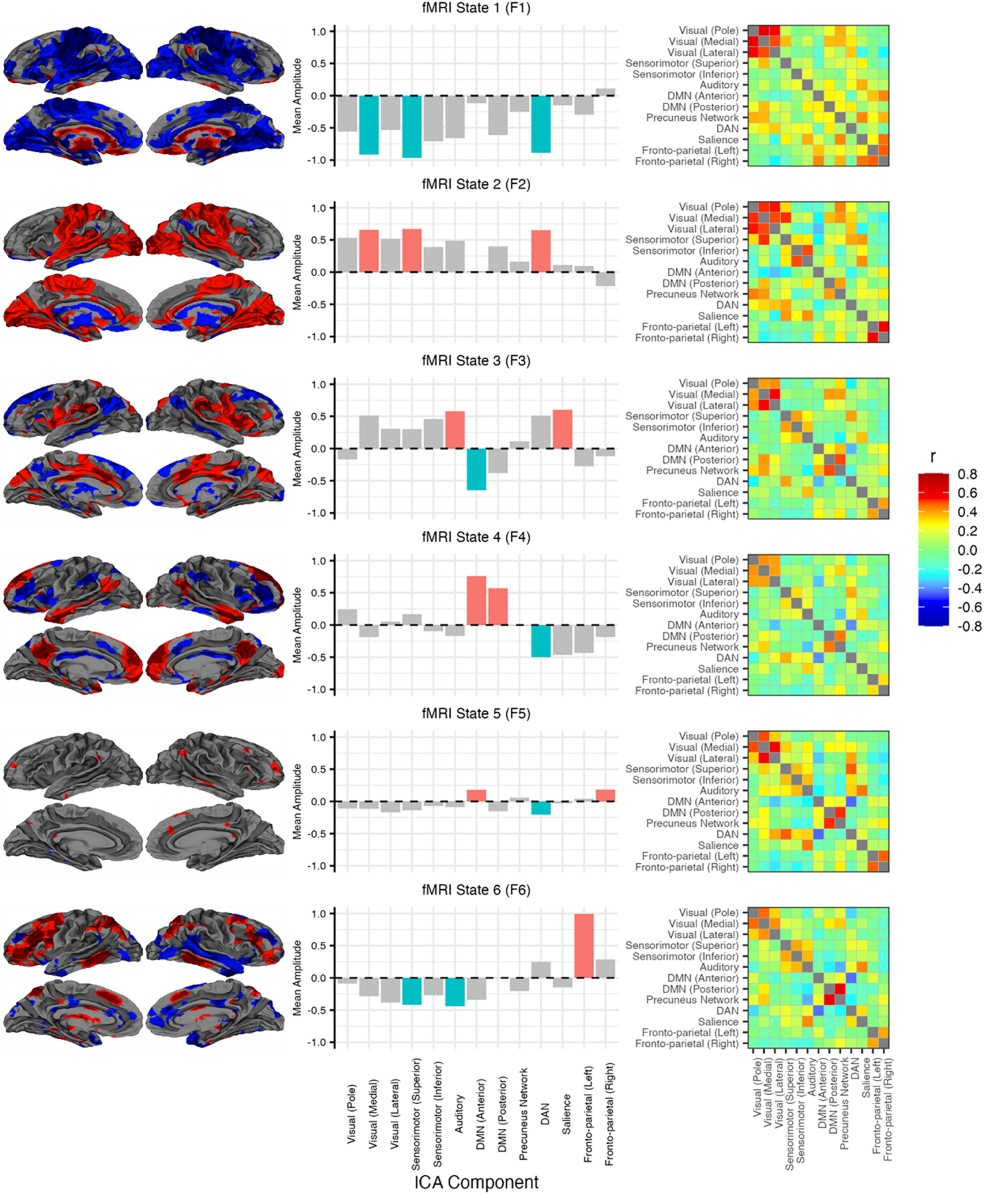
fMRI HMM state plots. Each row represents a different state identified by the HMM (F1 to F6). Column (a) shows the mean activation maps plotted on brain surfaces. Column (b) shows the corresponding bar chart for the mean activation maps, displaying the mean contribution of each IC to each HMM state; the coloured bars represent the three largest amplitudes (red: positive, blue: negative). Column (c) shows the estimated fc for each state. FC is estimated using the covariance matrix of each state. EEG, electroencephalography; FC, functional connectivity; fMRI, functional magnetic resonance imaging; HMM, hidden Markov model; IC, independent component.

**FIGURE 5:**
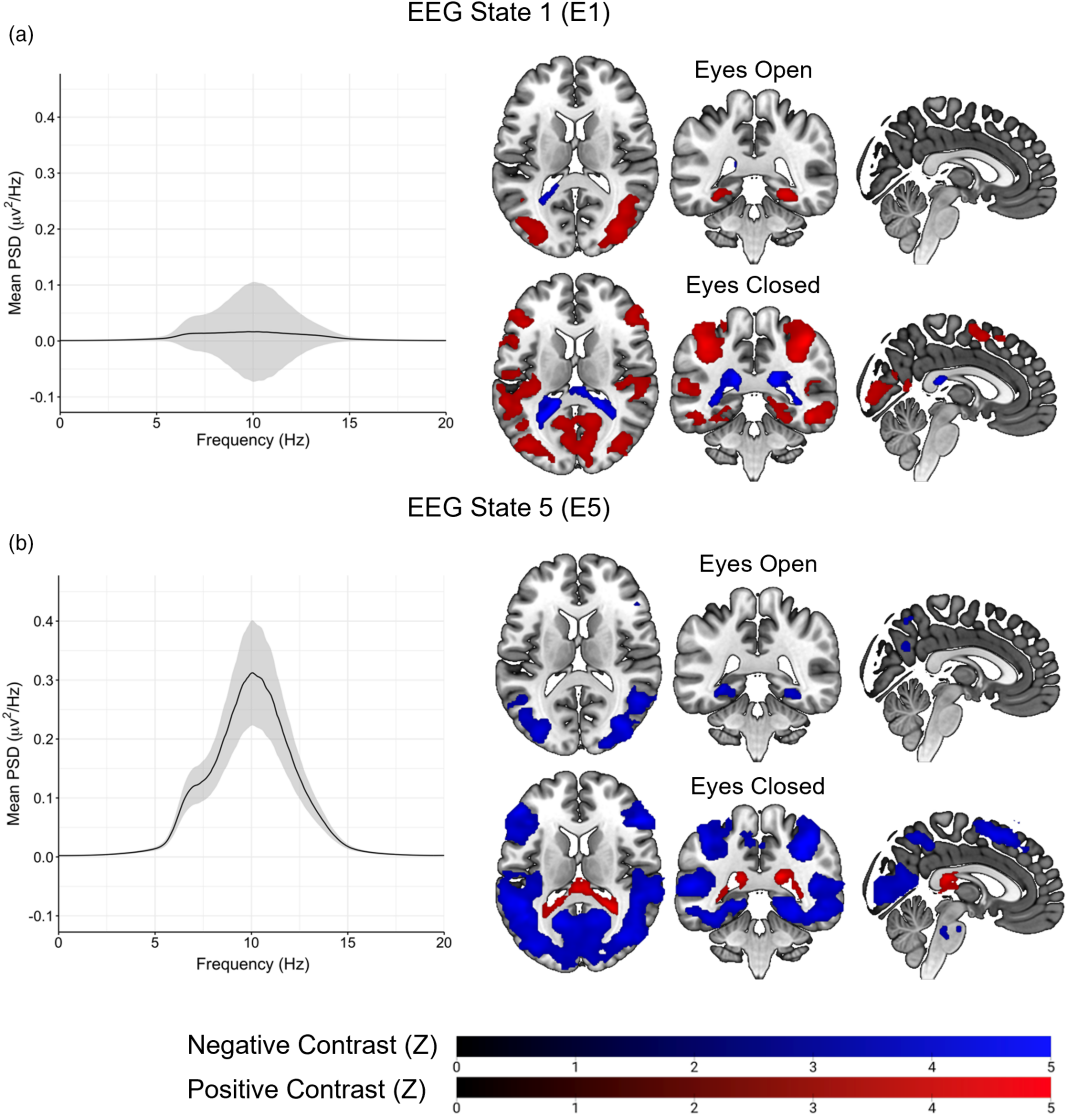
EEG HMM state spectral properties and voxel‐wise correlations between EEG HMM state probability time courses and the BOLD signal. (a) Power spectral density estimate and activation maps for state E2. (b) Power spectral density estimate and activation maps for state E5. Brain images plotted in MNI152 space (coordinates = 111 × 96 × 117). *Z* threshold = 3.1, cluster *p* threshold = 0.05. Images presented using neurological convention (left = left). Shaded regions represent 95% confidence intervals of the mean alpha power across the channels inputted to the HMM. EEG, electroencephalography; HMM, hidden Markov model.

The fMRI HMM identified a total of six states (labelled F1–F6), defined by the mean activity across the inputted networks and the FC between each network (see Figure 4). Observing the states, F1 and F2 each showed large activations/deactivations within multiple sensory regions, including sensorimotor, auditory, and visual cortex. The FC between visual regions was notably higher within these states relative to other states. F3 showed large activations within the salience network and sensory regions, particularly auditory, as well as a deactivation within the DMN. F4's largest activations were predominately within the DMN. F3 showed positive FC between medial visual cortex FC and the DMN and precuneus network, whereas F4's FC pattern on average resembled the other states, with overall the lowest FC strength of all the states. F5 exhibited very low activations across all networks relative to other states, with the DMN, right frontoparietal and DAN showing the largest de/activations. F6's largest activations were within a combination of the FPN and DAN, alongside deactivations within posterior somatosensory and auditory networks. Both F5 and F6 showed increased FC between the precuneus network and the DMN relative to other states. Five states were inferred with the EEG HMM (E1–E5), defined by changes in posterior alpha (7–13 Hz) power amplitude (Hilbert Transform) and alpha power correlations across the posterior electrodes (O1, O2, Oz, POz, PO3 and PO4). All five states primarily differed in mean amplitude, whereas showing similar patterns of covariance. State spectra were estimated using data from both conditions via a state‐wise weighted multi‐taper analysis for each channel and averaged. E5 exhibited the highest alpha power, followed sequentially by E4, E2, E3 and E1 which exhibited the lowest alpha power (see examples in Figure 5). Only states E1 and E5 showed significant correlations between their state probability time course and the BOLD signal (see Figure 5). Within the EC condition, E5 exhibited negative correlations across cortex, including visual cortex, parietal cortex, frontal cortex and the DAN. In contrast, during the EO condition, E5 exhibited negative BOLD correlations exclusively within the lateral visual cortex. Conversely, E1 displayed positive BOLD correlations with similar activation patterns to E5 during both EO and EC conditions. Unlike E5, the BOLD correlations during the EC condition were notably less widespread across the occipital and parietal cortex.

**FIGURE 6:**
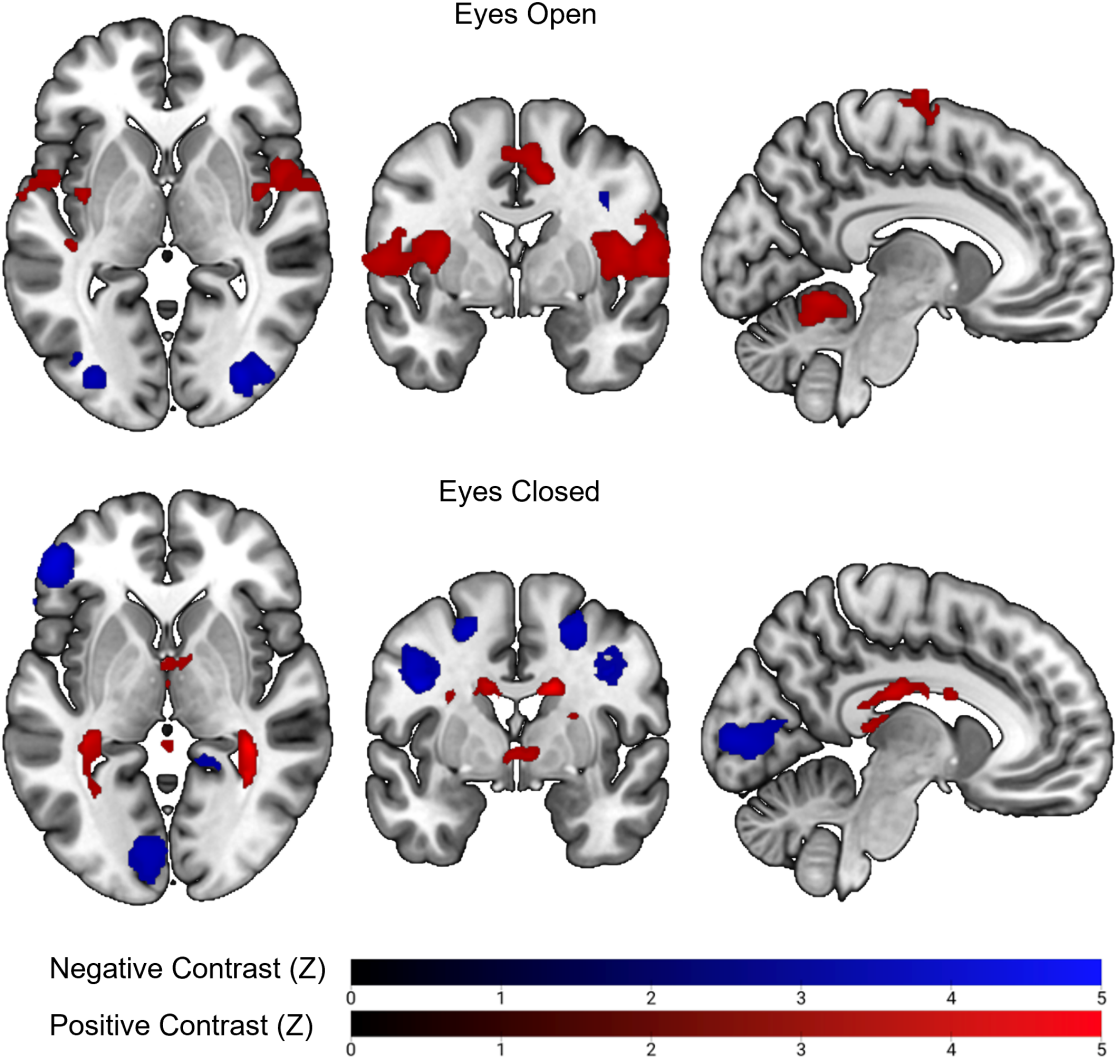
Group‐level statistical maps from the conventional alpha‐BOLD correlation approach plotted in MNI152 space (coordinates: 92 × 149 × 97). Mean positive (red) and negative (blue) correlations between alpha power and BOLD signal are shown for both EO (upper) and EC (lower) rest conditions. *Z* threshold = 3.1, cluster threshold = 0.05. Images presented using neurological convention (left = left). EC, eyes closed; EO, eyes open.

The conventional analysis, investigating alpha power correlations with BOLD signal, found similar results (see Figure 6). During the EC condition, negative alpha‐BOLD correlations were observed within primary visual cortex, frontal cortex and DAN. The correlations were notably less widespread compared to the E5 activation map but showed a large amount of overlap. Similarly, during the EO condition, negative alpha‐BOLD correlations were observed within lateral visual cortex, though notably less than observed for E5. However, unlike E5, positive alpha‐BOLD correlations were observed within the insula bilaterally, anterior cingulate cortex and the cerebellum during the EO condition.

### 3.4 Fractional occupancy sliding window analysis

**FIGURE 7:**
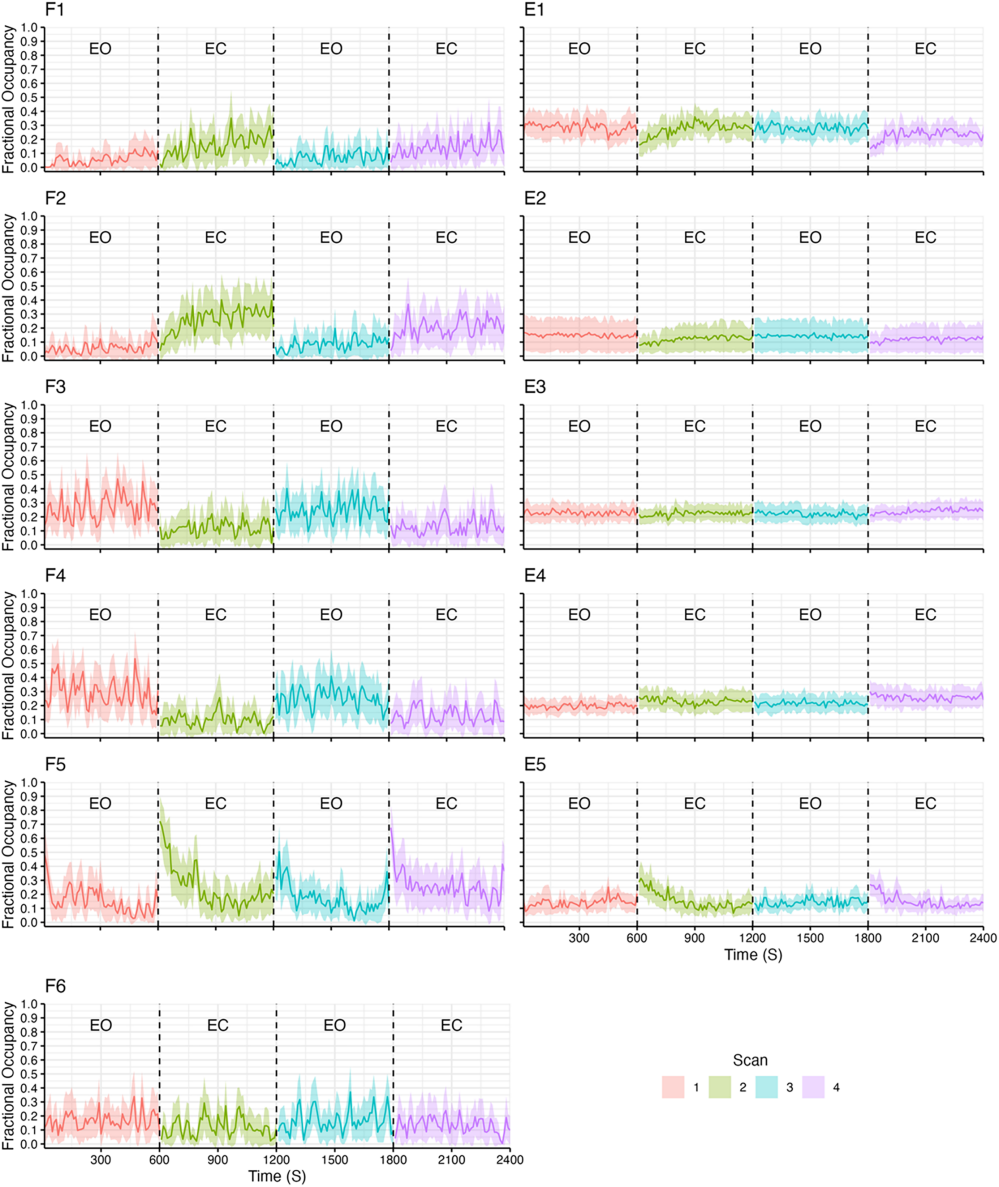
Time courses of state fractional occupancies across all four scans (in order of acquisition EO, EC, EO, EC), calculated using a sliding window analysis, for both HMMs. Window size = 10 s, overlap = 0. EC, eyes closed; EO, eyes open; HMM, hidden Markov model.

The sliding window analysis of the fractional occupancies revealed multiple unique patterns of state dynamics within the fMRI and EEG HMMs. Specifically, the fMRI sliding window analysis found that states F1 and F2 showed gradual increases in fractional occupancy upon eye closure, before decreasing to near zero upon eye opening (see Figure 7). States F3 and F4 both increased their occupancy during the EO conditions. F5 showed dramatic increases in fractional occupancy upon eye closure, before gradually returning to a baseline level. It was also noted that fractional occupancy of F5 increased upon reopening of the eyes, but to a lesser magnitude than upon eye closure. State F6 exhibited the most static time course of occupancy, with no notable changes between conditions (see Figure 7).

Regarding the EEG sliding window analysis, states E1, E2, E4 and E5 all showed changes upon eye closure, with the former two states showing a rapid decrease in fractional occupancy upon eye closure, and the latter two states exhibiting the opposite pattern (see Figure 7). All four states then gradually returned to a baseline level. State E3 showed minimal variation across the duration of the session, except for a minor increase within the final scan. State E4 showed a gradual increase across all four scans, with fractional occupancy increasing upon each eye closure.

**FIGURE 8:**
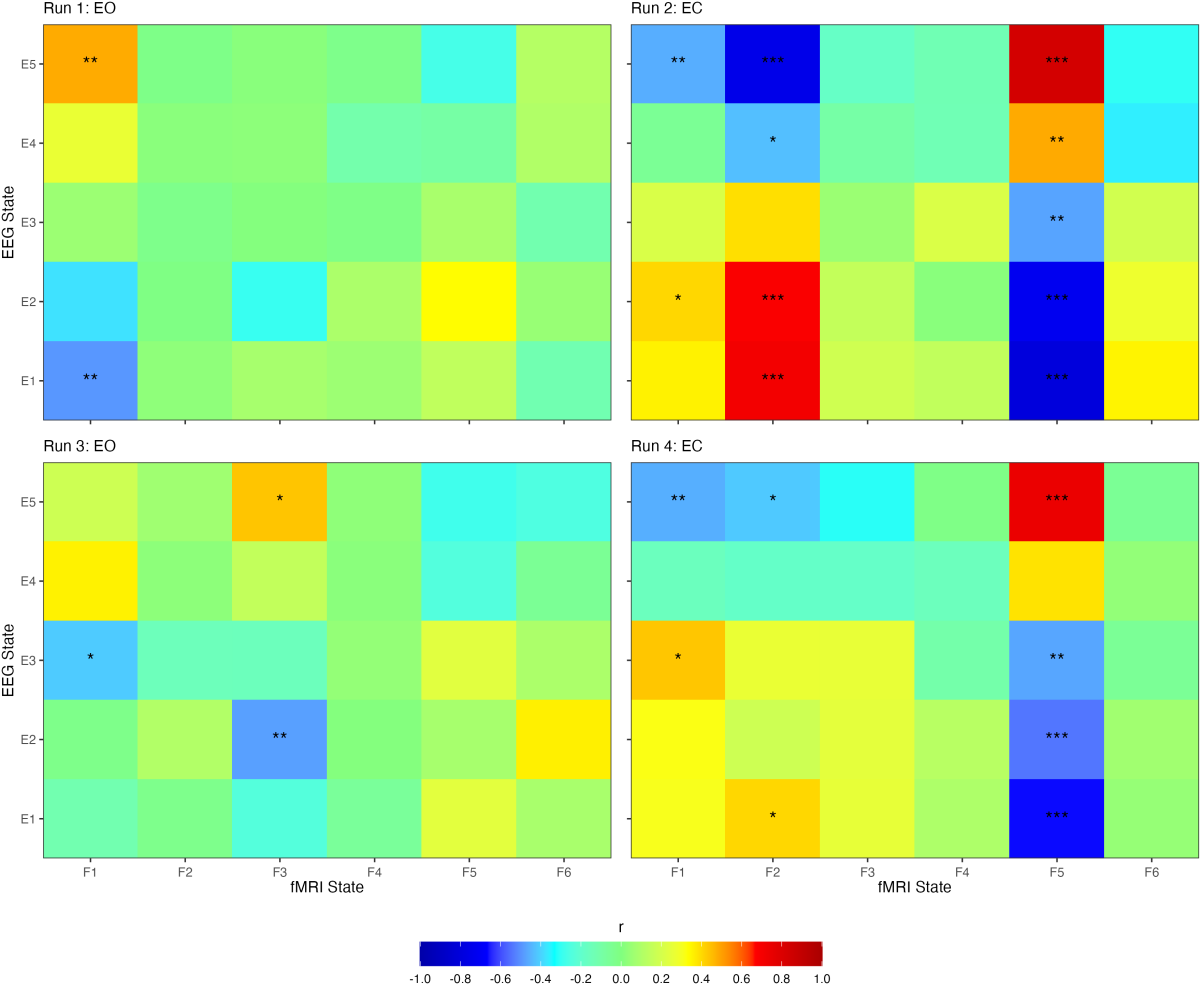
Correlation matrices of sliding window time courses across EEG and fMRI sliding window time courses. Pearson correlations were calculated at the group level. EC, eyes closed; EEG, electroencephalography; EO, eyes open; fMRI, functional magnetic resonance imaging. All *p* values are Bonferroni corrected (number of comparisons = 30). * = *p* < .05, ** = *p* < .01, *** = *p* < .001.

Correlating the sliding window time courses across the EEG and fMRI model states revealed strong correlations between several states (see Figure 8). Specifically, during run 1 (EO), state F1 exhibited a range of correlations with the EEG states, with state E1 showing the strongest negative correlation and E5 showing the strongest positive correlation. Similarly, during run 2, states F2 and F5 also showed strong correlations with the EEG states, with F2 showing strong positive correlations with states E1 and E2, and strong negative correlations with E5 and E4. Conversely, state F5 showed an inverse relationship with the EEG states, showing strong positive correlations with E5 and E4, and strong negative correlations with E1 and E2. Only low correlations between states were observed in run 3, with only state F3 showing a moderate positive correlation with state E5, and a moderate negative correlation with state E2. Finally, during run 4, state F5 showed similar correlations with the EEG states as during run 2. Additionally, states F1 and F2 all showed moderate positive correlations with states E1, E2 and E3, and moderate negative correlations with state E5.

### 3.5 State metric comparison

**FIGURE 9:**
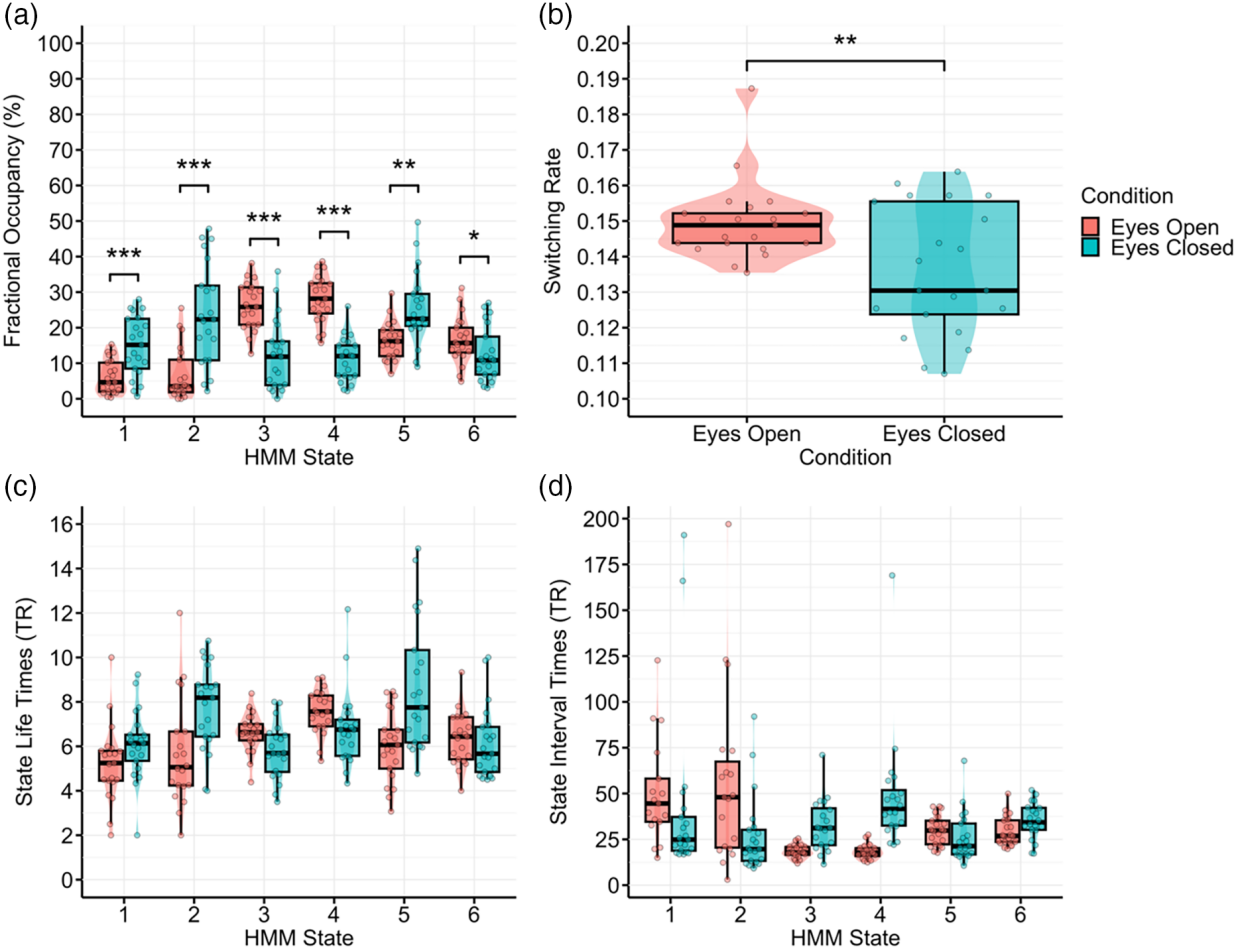
Comparison of fMRI HMM state metrics between EO and EC conditions. (a) Fractional occupancy. (b) Switching rate. (c) State life time. (d) State interval time. EC, eyes closed; EO, eyes open; fMRI, functional magnetic resonance imaging; HMM, hidden Markov model. All *p* values are Bonferroni corrected (number of comparisons = 6). * = *p* < .05, ** = *p* < .01, *** = *p* < .001.

Statistical comparison of the fMRI HMM state metrics across EO/EC conditions revealed multiple significant differences. Significant differences in state fractional occupancy between conditions were found for all states. F1, F2 and F5 had a significantly higher fractional occupancy during the EC condition, whereas F3 and F4 had a significantly higher fractional occupancy during the EO condition (see Figure 9a and Table 1). State lifetimes also showed differences between conditions, with F1, F2 and F5 having longer state lifetimes during the EC than the EO condition, and F4 having the opposite (see Figure 9c). All states had lower state interval times when in the conditions in which they had higher fractional occupancy (see Figure 9d). The state switching rate was significantly higher for the EO condition relative to the EC condition (difference = 0.01, 95% CI: [3.81e−03 to 0.02], *t*(20) = 2.90, *p* = .009; Cohen's *d* = 0.63, Cohen's *d* 95% CI: [0.16–1.10]) (see Figure 9b).

**TABLE 1:** Paired *t* test parameters for fMRI HMM state fractional occupancy comparisons between the EO and EC conditions.

HMM state	Difference	*t*(20)	p	Adjusted *p*	95% CI	Cohen's *d*	Cohen's *d* 95% CI
F1	−0.09	−6.88	<.001	<.001	[−0.11 to −0.06]	−1.50	[−2.12 to −0.86]
F2	−0.17	−5.82	<.001	<.001	[−0.23 to −0.11]	−1.27	[−1.84 to −0.68]
F3	0.14	6.55	<.001	<.001	[0.09–0.18]	1.43	[0.81–2.04]
F4	0.17	9.74	<.001	<.001	[0.13–0.21]	2.13	[1.34–2.90]
F5	−0.09	−4.22	<.001	.003	[−0.14 to −0.05]	−0.92	[−1.43 to −0.40]
F6	0.04	3.46	<.001	.015	[0.02–0.06]	0.75	[0.26–1.23]

*Note*: *p* values adjusted using the Bonferroni method (number of comparisons = 6).

Abbreviations: EC, eyes closed; EEG, electroencephalography; EO, eyes open; fMRI, functional magnetic resonance imaging; HMM, hidden Markov model.

**FIGURE 10:**
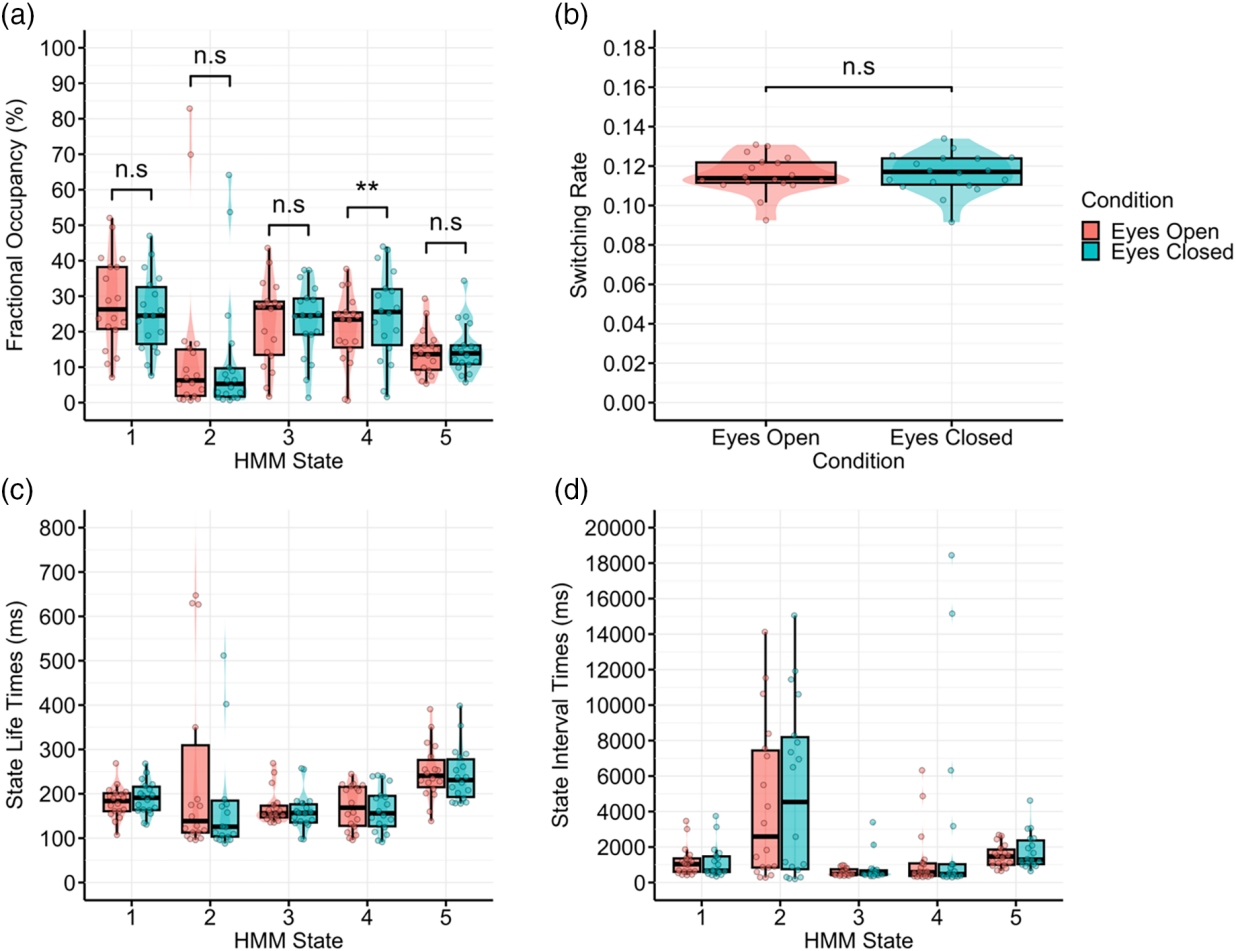
Comparison of EEG HMM state metrics between EO and EC conditions. (a) Fractional occupancy. (b) Switching rate. (c) State lifetime. (d) State interval time. EEG, electroencephalography; HMM, hidden Markov model. All *p* values are Bonferroni corrected (number of comparisons = 6, ** = *p* < .01, n.s = non‐significant).

In comparison, only one significant difference in state metrics was found between the two conditions for the EEG HMM (see Figure 10a, b, and Table 2). State E4 had a significantly higher fractional occupancy during the EC condition relative to the EO condition. No significant difference in switching rate was found between the two conditions (difference = −6.63e−04, 95% CI: [−2.23e−03 to 9.02e−04], *t*(17) = −0.89, *p* = .384; Cohen's *d* = −0.21, Cohen's *d* 95% CI: [−0.67 to 0.26]). State lifetimes were highest for states E1 and E5, and lowest for E4. Both states E4 and E5 showed a minor increase in state lifetimes when within the EC rest condition relative to the EO rest condition (see Figure 10c). Interval times also showed minimal variance between the two conditions, with E1 and E2 showing a minor reduction during the EO rest condition, and E3 showing a minor increase during the EO rest condition (see Figure 10d).

**TABLE 2:** Paired *t* test parameters for EEG HMM state fractional occupancy comparisons between the EO and EC conditions.

HMM state	Difference	*t*(17)	*p*	Adjusted *p*	95% CI	Cohen's *d*	Cohen's *d* 95% CI
E1	0.03	2.06	.055	.277	[0.00–0.06]	0.48	[−0.01 to 0.97]
E2	0.03	1.38	.187	.934	[−0.01 to 0.06]	0.32	[−0.15 to 0.79]
E3	<0.01	−0.52	.610	1	[−0.04 to 0.02]	−0.12	[−0.58 to 0.34]
E4	−0.04	−4.49	<.001	.002	[−0.06 to −0.02]	−1.06	[−1.63 to −0.47]
E5	−0.01	−1.15	.268	1	[−0.03 to 0.01]	−0.27	[−0.74 to 0.20]

*Note*: *p* values adjusted using the Bonferroni method (number of comparisons = 5).

Abbreviations: EC, eyes closed; EEG, electroencephalography; EO, eyes open; fMRI, functional magnetic resonance imaging; HMM, hidden Markov model.

Based upon the results of the sliding window analysis (i.e., time courses in Figure 7), the state metrics were additionally compared solely within the first quarter of each scan (2.5 mins), since various states demonstrated larger changes within that period. Within the EEG HMM, this revealed significant differences in the fractional occupancies of state E5 (difference = −0.09, 95% CI: [−0.15 to −0.03], *t*(17) = −3.32, *p* = .004, adjusted *p* = .020; Cohen's *d* = −0.78, Cohen's *d* 95% CI [−1.30 to −0.24]) between conditions. The switching rate did not differ significantly between conditions during the first quarter. Conversely, for the fMRI analysis, the significance of both state fractional occupancy and switching rates did not change when restricted to the first quarter.

## 4 DISCUSSION

### 4.1 Summary

This study applied HMM to EEG‐fMRI data to investigate changes in brain state dynamics during 10‐min scans of either EO or EC resting state. HMMs were trained separately on each imaging modality and underwent model selection to determine the optimal number of states (fMRI = 6, EEG = 5). A comparison of the state metrics found significant differences in the fractional occupancy of both the fMRI and EEG states. A sliding window analysis further revealed that specific fMRI and EEG states showed substantial changes in fractional occupancy upon eye closure, before returning to baseline level after approximately two and half minutes. Additionally, the sliding window analysis revealed states from the fMRI model showed strong correlations in fractional occupancy with states from the EEG model, indicating that states inferred from different modalities were capturing similar temporal dynamics. Finally, a GLM analysis revealed that state E5, which was associated with the highest alpha power, showed BOLD correlations closely resembling canonical alpha‐BOLD correlations.

### 4.2 fMRI HMM states

The fMRI HMM identified six states, each with unique activation maps. Of these, F1, F2 and F5 showed significantly increased fractional occupancy during the EC rest, whereas states F3 and F4 showed decreased fractional occupancy during EC. With the exception of F3, all identified states have been reported within either previous HMM research (Song et al., 2023) and/or complex PCA resting‐state research (Bolt et al., 2022), indicating that these states occur reliably. Furthermore, the component equivalent to states F1 and F2 identified within this study was highly correlated with the global BOLD signal (Bolt et al., 2022). Though the origin and significance of the global BOLD signal have been heavily debated, particularly in regard to whether it is a representation of noise or physiologically plausible signal (Liu et al., 2017), animal literature has demonstrated that it correlates strongly with local field potentials, but only during periods of eye closure (Schölvinck et al., 2010). This suggests that the global BOLD signal exhibits different dynamics within EO and EC rest, a finding that has been reported within previous fMRI literature (Agcaoglu et al., 2019; Weng et al., 2020), with research demonstrating that BOLD signal amplitude shows significantly higher variability within the EC condition (Jao et al., 2013). Given our results this would seem unsurprising, given that both states were defined by large changes in activity within sensory regions, as well as showing greatly increased FC with eye closure. Furthermore, the reported increase in variability during the EC condition, matches the results here, with the state fractional occupancy values exhibiting higher variability during the EC condition relative to the EO condition. As to what these states represent, previous research demonstrating increases in BOLD signals within sensory regions during eye closure has suggested that this represents mental imagery (Marx et al., 2004). Alternatively, given the states' potential association with the global BOLD signal, they could represent a reduction in vigilance, given previous EEG‐fMRI research finding correlates between the global BOLD signal and vigilance measures (Wong et al., 2016). It has also been suggested that EC resting‐state scans contain periods of sleep (Tagliazucchi & Laufs, 2014) and future work will be needed to understand the impact of sleep on HMM states and their properties.

Regarding the remaining states, the interpretation is less complex. State F4 solely consisted of the activity within the DMN and showed a higher fractional occupancy during the EO resting condition, as previously reported (Jao et al., 2013; Wang et al., 2022; Yan et al., 2010). This potentially occurs as a result of the removal of visual stimuli when the eyes are closed, as both posterior cingulate cortex and medial prefrontal cortex have been linked to sensory monitoring (Brandman et al., 2021; Raichle et al., 2001). State F3, on the other hand, primarily consisted of large activations within the salience and auditory networks, with the same pattern of fractional occupancy as state F4. As the salience network is primarily associated with the detection of sensory stimuli (Downar et al., 2000), it can be hypothesised that the state was also more active during EO rest as result of being able to perceive external visual stimuli. Finally, state F4 exhibited minimal activation across all networks relative to the other states. However, the sliding window analysis revealed that F5 was the only fMRI state to show an increase in fractional occupancy at eye closure. Additionally, the state also showed a similar increase in functional connectivity between visual regions as in states F1 and F2. Thus, the state appears to represent the initial increase in FC within the visual cortex upon eye closure. Together, this suggests that the fMRI HMM was able to accurately identify the changes in dynamics between the two rest conditions.

### 4.3 EEG HMM states

The EEG HMM identified five states with varying alpha power, with E5 exhibiting the highest alpha power, followed by states E4, E2, E3 and E1. Using the state time courses from each state as regressors within an fMRI GLM analysis, significant BOLD correlates were identified for both the highest (E5) and lowest (E1) alpha power states in both EO and EC conditions. These activation maps closely resembled the activation maps obtained from the conventional alpha‐BOLD approach. Specifically, during the EC condition, state E5 showed widespread negative correlations across occipital and parietal cortex, as well as within the DAN. Conversely, during the EO condition, state E5 showed bilateral negative correlations within lateral occipital cortex. This suggests that the HMM approach can identify states whose time course of fractional occupancy has meaningful BOLD correlates, resulting in brain states with both high temporal and spatial properties.

Though neither E1 nor E5 showed a significant difference in fractional occupancy across the two conditions, the sliding window analysis revealed that E5 showed a significant spike in fractional occupancy during the first quarter (2.5 min) of each EC scan, before gradually returning to a baseline level. This pattern in fractional occupancy appeared unique to the EEG HMM states, with fMRI state fractional occupancy remaining stationary across the entire duration of each scan (except for F4). We hypothesise that this is due to the fMRI states representing slow global metabolic changes over time, whereas the EEG states solely represent more dynamic changes in alpha power. This is supported by the fact that E5's fractional occupancy time course closely resembled the changes in alpha power observed in the alpha power time courses, suggesting that state E5 specifically represents the initial change in alpha power induced by eye closure. Furthermore, when restricting the fractional occupancy to the first quarter of each scan, state E5 showed a significant increase in fractional occupancy during the EC condition, providing further support for this theory.

### 4.4 fMRI‐informed EEG state spatial properties

By utilising the concurrently collected fMRI data, we were able to infer the spatial properties of the EEG states E5 and E1, which showed high similarity to maps of alpha‐BOLD correlations obtained from conventional EEG‐fMRI (de Munck et al., 2007; Goldman et al., 2002; Laufs et al., 2006; Mayhew & Bagshaw, 2017). Interestingly, the identified BOLD maps for the two states were almost identical inverses of each other. One reason this potentially occurred is that E5 was the state associated with high alpha power activity, whereas E1 was associated with the low alpha power activity. Thus, when one state was more active, the other was likely to be inactive, resulting in inverse BOLD maps. Overall, these results are in accordance with previous literature (Hunyadi et al., 2019), and demonstrate that fMRI data can be used to infer the spatial activity of states defined by EEG data, resulting in states with both high spatial, temporal, and potentially spectral resolution (although in the current case this was restricted to the alpha band).

However, unlike previous literature, reliable BOLD maps were able to be identified from states that were only active for a short duration of the overall scan (~15%). This suggests that BOLD correlates obtained from conventional EEG‐fMRI approaches are predominantly driven by short periods of high alpha power, or ‘bursts’, as opposed to a sustained alpha rhythm that spans across the duration of an entire scan (Quinn et al., 2019). A similar conclusion has also been drawn from MEG data, with HMMs identifying beta ‘bursts’ during a motor task (Seedat et al., 2020).

### 4.5 Cross modality correlations

Correlating the sliding window time courses across modalities revealed that several states showed strong temporal correlations across modalities. This can be interpreted as showing that during a time point when the probability of being in a specific fMRI state was high across the subject group, then it was likely that those subjects were also in a specific EEG state. Most notably, states E5 and F5 showed a strong positive correlation during the EC runs, with their fractional occupancy beginning high and steadily decreasing back to baseline after approximately 2.5 min. This correlation is especially interesting due to state E5 showing the highest alpha power of the EEG states, thus indicating that the dynamics of fMRI state F5 closely resemble the slow temporal changes in alpha power. However, it is difficult to interpret this relationship further, given that F5 showed low mean BOLD activity across all networks relative to the other fMRI states. Despite this, these correlations in temporal dynamics across neuroimaging modalities indicate that the states are capturing similar underlying dynamics in the data.

### 4.6 Limitations and future work

This study had several limitations. First, the study did not employ counterbalancing, with all participants completing the scans in the same order. This makes it difficult to discuss any links to vigilance as it is not possible to rule out potential order effects, and therefore counterbalancing of scan order should be employed in future research. Second, the EEG analysis was restricted to occipital alpha power. This analysis was chosen due to a priori knowledge of how occipital alpha power changes upon eye closure; however, it only serves as a limited definition of a brain state. In future work, a more complex observation model, such as a time‐delay embedded (TDE) model (Vidaurre, Hunt, et al., 2018) or multivariate autoregressive (MAR) model (Vidaurre et al., 2016), should be employed to directly infer states according to their spectral properties, including both power and phase. However, it is yet unclear how well these observation models will perform on concurrent EEG‐fMRI data, given the restricted bandwidth available due to the residual harmonics of the gradient and BCG artefacts. It is also acknowledged that the HMM approach itself is not free of limitations. The primary limitation being that it is reliant on the Markov assumption, assuming that the probability of being in any one state at a time point is determined solely based on the state active at the time point prior. Additionally, the HMM approach requires an extensive model selection process to ensure both the reliability and validity of the mode. Despite these limitations, the results here suggest that the HMM approach can detect dynamic changes in both the BOLD signal and alpha power that are in accordance with the literature.

## 5 CONCLUSIONS

This study identified unique spatiotemporal dynamics during periods of EO and EC rest in concurrent EEG‐fMRI recordings. In line with previous research, the fMRI model found significant changes in the mean activity and functional connectivity of sensory and attention networks, whereas the EEG model identified the canonical increase in alpha power upon eye closure. This demonstrates that the HMM approach is capable of reliably identifying brain state dynamics in EEG‐fMRI data. Furthermore, we demonstrate that multimodal data can be used help to infer the spatial properties of EEG‐defined states using the concurrently recorded fMRI data, resulting in states with high spatial and temporal resolution. Specifically, we found that the high alpha state showed significant BOLD activation patterns resembling those identified in the literature. Given the low fractional occupancy of the state (~15%), this suggests that these BOLD patterns are driven by short periods of high‐intensity alpha, as opposed to a sustained rhythm. Finally, we found that several states showed a strong correlation in fractional occupancy across modalities, indicating that states defined by EEG and fMRI data capture similar dynamics despite differences in spatial and temporal resolution. However, it is noted that the observation model used with the HMM provides an oversimplification of the dynamics, given that it can only model changes in power within a predefined frequency band, and thus future research should utilise more advanced observation models, such as the TDE (Vidaurre, Abeysuriya, et al., 2018; Vidaurre, Hunt, et al., 2018) or MAR (Vidaurre et al., 2016) models to improve the spectral definitions of the EEG states. Overall, these findings demonstrate the efficacy of applying HMM to multimodal data for the purposes of brain state analysis, the ability of multimodal data to improve the characterisation of brain states, and show that state dynamics often show strong temporal correlations across modalities.

## AUTHOR CONTRIBUTIONS

Brandon T. Ingram: Data curation; formal analysis; roles/writing—original draft. Stephen D. Mayhew and Andrew P. Bagshaw: Funding acquisition; supervision; writing—review and editing.

## CONFLICT OF INTEREST STATEMENT

The authors declare no conflicts of interest.

## Data Availability

De‐identified participant data can be made available by contacting the corresponding author. Reuse is only permitted following written agreement from the corresponding author and Primary Institution.
